# Adipose tissue: a new target for electroporation-enhanced DNA vaccines

**DOI:** 10.1038/gt.2017.96

**Published:** 2017-12-07

**Authors:** P D Fisher, C J Brambila, J R McCoy, W B Kiosses, J M Mendoza, J Oh, B S Yung, K Schultheis, T R F Smith, K E Broderick

**Affiliations:** 1 Inovio Pharmaceuticals, Inc., San Diego, CA, USA; 2 The Scripps Research Institute, Core Microscopy Facility, La Jolla, CA, USA

## Abstract

DNA vaccines delivered using electroporation (EP) have had clinical success, but these EP methods generally utilize invasive needle electrodes. Here, we demonstrate the delivery and immunogenicity of a DNA vaccine into subcutaneous adipose tissue cells using noninvasive EP. Using finite element analysis, we predicted that plate electrodes, when oriented properly, could effectively concentrate the electric field within adipose tissue. In practice, these electrodes generated widespread gene expression persisting for at least 60 days *in vivo* within interscapular subcutaneous fat pads of guinea pigs. We then applied this adipose-EP protocol to deliver a DNA vaccine coding for an influenza antigen into guinea pigs. The resulting host immune responses elicited were of a similar magnitude to those achieved by skin delivery with EP. The onset of the humoral immune response was more rapid when the DNA dose was spread over multiple injection sites, and increasing the voltage of the EP device increased the magnitude of the immune response. This study supports further development of EP protocols delivering gene-based therapies to subcutaneous fat.

## Introduction

The delivery of DNA-encoded vaccine antigens is a promising vaccination strategy that offers several advantages over traditional vaccine methods. Plasmid DNA production can be both rapid and cost effective, and the product may not require cold chain. Because of the plasticity of DNA-based constructs, a plasmid can be specifically designed to code for any known antigen as long as the sequence of the pathogen is known. Unlike viral vector platforms, there is no vector-acquired immunity. This permits repeated administrations of plasmid DNA vaccines to boost immunity, without the requirement to re-engineer the construct for each vaccine. Before the employment of electroporation (EP) as an enabling technology, the success of DNA vaccines has historically been limited because transfection efficiency is poor following simple injection of naked DNA. Overcoming this barrier by increasing the dose is impractical when scaling up to larger animals and humans, and several clinical trials in the past utilizing protocols based on injection of naked plasmid DNA failed to replicate the promising preclinical data.^1, 2, 3^ To overcome these delivery limitations, the physical application of EP has been established as a key enabling technology for the *in vivo* delivery of plasmid DNA vectors for vaccination.

To perform *in vivo* EP, brief electrical pulses are applied at the site of drug injection, causing transient permeability of cell membranes because of the electric field generated in the targeted tissue. This permeability allows up to 1000-fold increases in transfection efficiency.^4, 5^ DNA vaccines delivered using EP can lead to antibody responses comparable to leading viral vectors.^6, 7, 8^ Furthermore, EP-enhanced DNA vaccines have been shown to drive strong cellular immune responses in large animals and humans that can be essential for vaccines against chronic infections and for cancer immunotherapies.^9, 10, 11, 12^ Recently, a therapeutic DNA EP vaccine for human papilloma virus was shown to be effective at reducing or eliminating the precancerous lesions and viral loads in patients with human papilloma virus-related cervical intraepithelial neoplasia.^13^ This was the first randomized controlled trial demonstrating the clinical efficacy of an EP-enhanced DNA vaccine.

Traditionally, EP-enhanced DNA vaccinations are performed intramuscularly (IM-EP) using needle electrodes that are inserted and pulsed at the injection site. Alternatively, intradermal EP (ID-EP) is a less invasive procedure than IM-EP because of shallower penetration and smaller-gauge needles required, and can also promote a potent humoral and cellular immune response.^14, 15, 16, 17^ IM-EP is an invasive procedure, but is capable of delivering large volumes of DNA (∼1 ml) and generating long-term gene expression, whereas ID-EP delivers smaller volumes (∼50–100 μl) but targets a more accessible tissue enriched with resident immune cells.

Clinically, the subcutaneous injection route is popular because it is generally more tolerable than intramuscular injection, technically simple to administer and suitable for efficacious delivery of many drugs, including biologics and vaccines.^18^ Therefore, as an alternative to IM-EP and ID-EP, we sought to investigate subcutaneous adipose tissue as a potential target for EP-enhanced plasmid DNA vaccine delivery by developing a noninvasive adipose-EP method and evaluating its gene expression and immunogenicity. Adipose, or fat, tissue is present in a layer dividing the skin and skeletal muscle. Adipose tissue is a loose connective tissue composed predominantly of adipocytes held together by extracellular matrix proteins. The primary role of fat is to store energy in the form of lipids and act as a cushion to insulate the body. However, far from being an inert storage tissue, adipose has also been shown to be a major endocrine organ, responsible for hormone production and secretion.^19, 20^ In addition, fat also has immune-modulatory functions, and recent research has shown that macrophages within adipose tissue, or even adipocytes themselves, can function as antigen-presenting cells.^21, 22^ In the case of EP-enhanced drug delivery, subcutaneous adipose tissue can act as an electrical barrier that must be physically bypassed using needle electrodes for IM-EP, and has historically been ignored as a potential target tissue for these treatments. ID-EP DNA vaccinations have been shown to transfect some adipocytes within the hypodermis, but the predominant transfected cell types for these treatments are in the dermis and epidermis.^23^ To date, the role that adipose tissue itself plays in DNA vaccines remains unexplored. The motivation of the present study is to evaluate a new method of DNA EP vaccination that preferentially targets adipocytes within subcutaneous fat tissue. First, finite element analysis was used to demonstrate that noninvasive plate electrodes can be used to generate electric fields within subcutaneous adipose tissue in a highly specific manner compared with invasive needle EP. Next, a guinea pig model was used to evaluate spatial distribution of transfected adipocytes and kinetics of gene expression within adipose tissue following noninvasive adipose-EP. Finally, the humoral and cellular immunogenicity of several adipose-EP protocols was compared with ID-EP in guinea pigs.

## Results

### Finite element analysis

**Figure 1:**
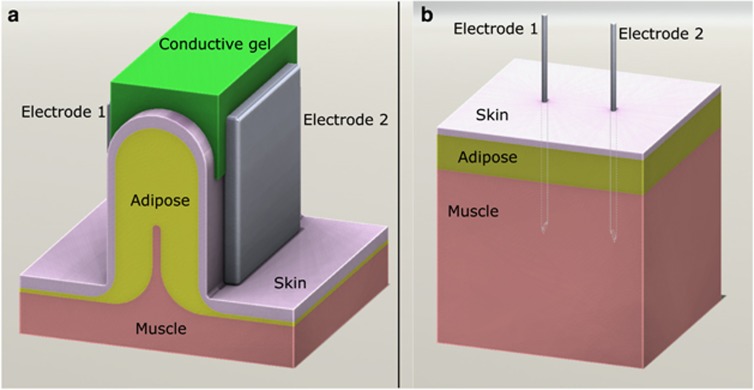
Three-dimensional (3D) computer models of tissue–electrode assemblies. (**a**) Noninvasive EP, with tissue folded between two plate electrodes. Distance between electrodes is 12 mm and electrode plate faces measure 20 mm × 20 mm. (**b**) Invasive EP using parallel needle electrodes inserted directly into tissue. Needles were 22-gauge with an insertion depth of 18 mm and a 10 mm inter-electrode gap. In both models, tissue is composed of three layers: 1 mm skin, 5 mm adipose and underlying muscle.

**Figure 2:**
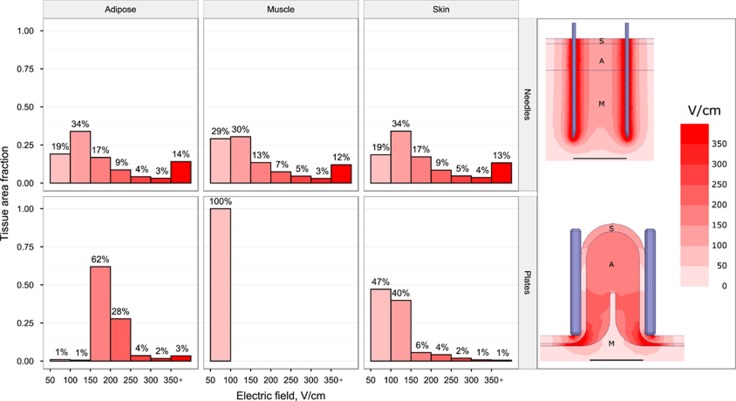
Simulated electric field distribution within different tissue types for needle (top) and plate (bottom) electrode configurations using a 200 V excitation voltage for each case. The histograms (from left to right: adipose, muscle, skin) quantify the electric field distribution within each tissue type for electric fields above 50 V cm^−1^. The images on the right show spatial electric field distributions for each configuration, with outlines and labels for skin (S), adipose (A) and muscle (M). Scale bar is 10 mm.

In a bid to understand the electrical properties of adipose tissue from an EP perspective, finite element analysis was carried out to quantify the predicted electric field distribution within each tissue type of interest (in this case, skin, muscle and adipose) using the two electrode designs—needles within flat tissue and plates around folded tissue—illustrated in Figure 1, using a 200 V excitation in both cases. Standard needle electrodes resulted in an electric field gradient distributed equally through skin, adipose and muscle (Figure 2, top), with the strongest electric fields at the electrode surface. In comparison, plate electrodes generated a more uniform electric field almost exclusively within adipose tissue (Figure 2, bottom), with comparatively small electric fields in skin and muscle. Needle electrodes were predicted to provide field strengths higher than 350 V cm^−1^ to 12–14% of each tissue, and fields lower than 150 V cm^−1^ to approximately half of treated tissue. Plate electrodes were predicted to produce electric fields between 150 and 350 V cm^−1^ in 95% of the treated adipose tissue. Meanwhile, muscle received no electric field above 100 V cm^−1^ and 87% of skin received less than 150 V cm^−1^ using the plate electrode design. Based on the results of these simulations, subsequent adipose-EP experiments utilized plate electrodes with voltages up to 200 V.

### Dye injection studies

**Figure 3:**
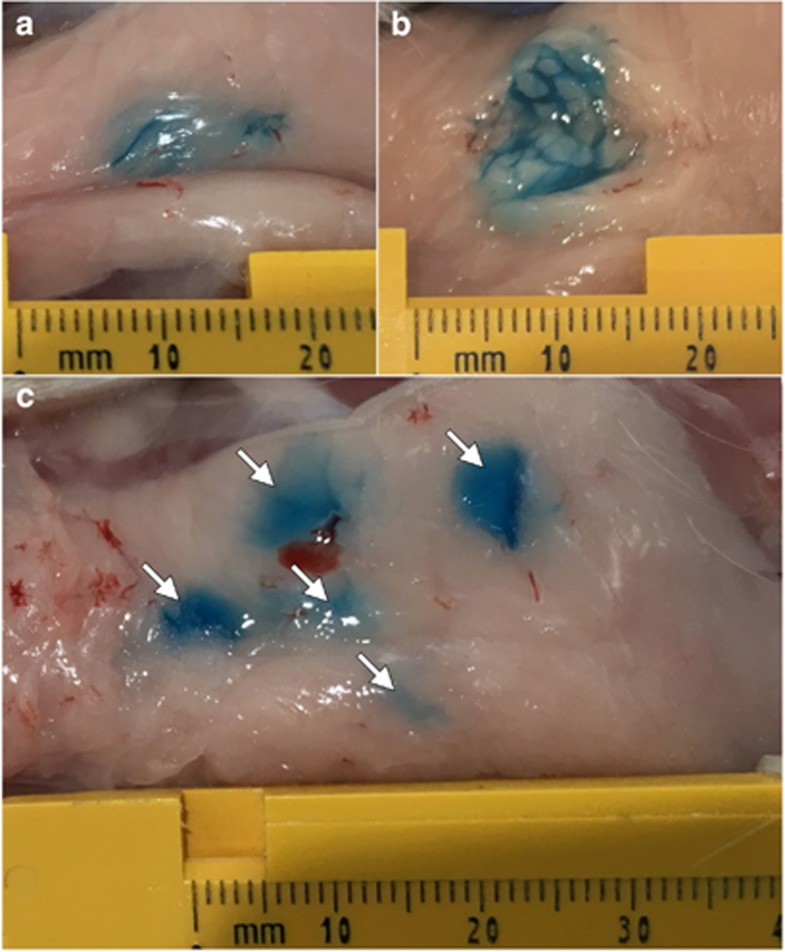
Dye injection into guinea pig subcutaneous fat pad. (**a**) Intact fat pad after a single, 100 μl injection. (**b**) Single site injection sectioned along sagittal plane to show fluid distribution within tissue. (**c**) Intact fat pad after five 50 μl injections. Arrows indicate injection sites.

Although the fluid dynamic properties of a bolus IM or ID injection are well characterized, the distribution of a fluid within *in vivo* subcutaneous fat was less clear. In addition, the impact of the physical effect of compressing the injection site between plate electrodes was unknown. To investigate these dynamics, dye injection studies were carried out in guinea pigs to allow visualization of the distribution of injected fluid within fat. After subcutaneous injection and squeezing between calipers, dye was visible within intact fat pads as an elongated bolus shape (Figure 3a). Upon dissection, dye appeared primarily within the collagenous septa dividing adipose lobes (Figure 3b). The blue stain was retained within the fat pad, with little or no stain present in the overlying skin or the underlying muscle (not shown). We performed the same dye analysis using multiple injection sites with the objective of increasing the distribution of DNA throughout the adipose tissue. When five separate 50 μl dye injections were performed and then clamped between electrode plates, five individual dye sites remained visible, although some were more prominent than others (Figure 3c). When dissected, each individual injection sites possessed similar dye distribution within the adipose tissue, with dye concentrated along the collagenous septa (not shown).

### *In vivo* transfection of adipocytes

**Figure 4:**
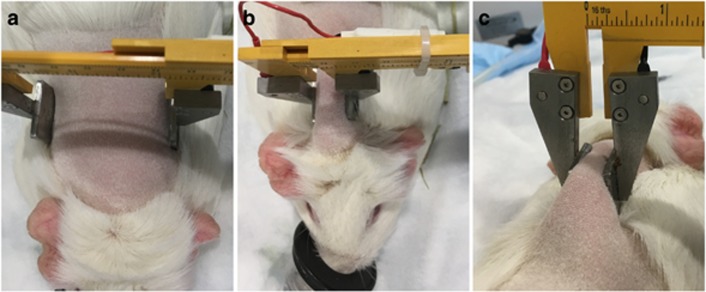
Adipose-EP procedure, showing (**a**) shaved interscapular region before application of electrodes, (**b**) the treatment site gripped between two noninvasive plate electrodes and (**c**) a back view of the gripped treatment site.

To assess the *in vivo* expression of a reporter construct in adipose tissue, 50 μg of plasmid coding for green fluorescent protein (GFP) was injected into guinea pig fat pads and electroporated with voltages ranging from 50 to 200 V using noninvasive plate electrodes as described in ‘General adipose-EP treatment procedure’ section. The treatment site and EP clamping procedure is shown in Figure 4.

**Figure 5:**
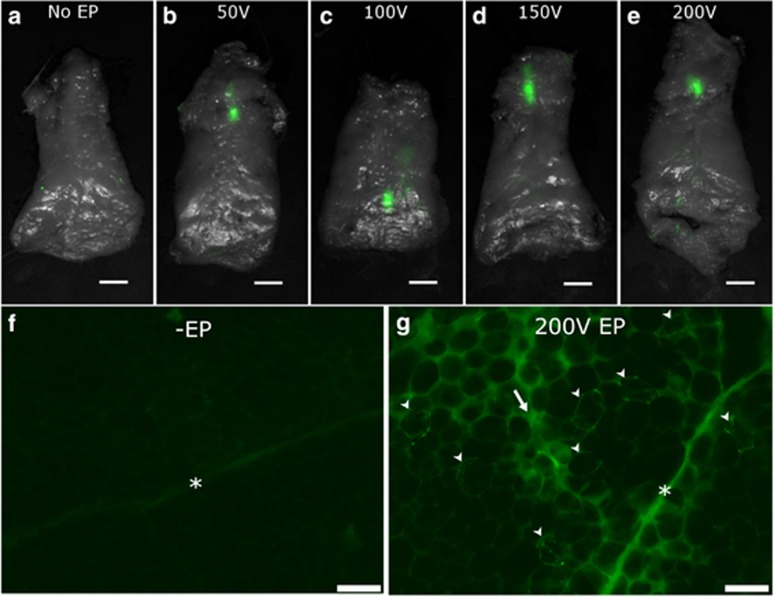
(**a**–**e**) GFP reporter construct expression (green) distribution throughout intact guinea pig fat pads following noninvasive adipose-EP ranging from 50 V (**b**) to 200 V (**e**) with dorsal surface visible. (**f**, **g**) Histological comparison of fluorescent signal at treatment site for guinea pigs receiving plasmid DNA injection without EP (**f**) or with 200 V adipose-EP (**g**). Markers indicate collagen septa (*), GFP-expressing adipocytes (arrowheads) and regions of high autofluorescence (arrow). Scale bars are 10 mm (**a**–**e**) or 100 μm (**f**, **g**).

At 3 days after performing these treatments, intact fat pads were removed and imaged at the gross tissue level. No GFP expression was detected in animals receiving plasmid injection without adipose-EP (Figure 5a). In adipose-EP-treated fat pads, GFP was expressed exclusively at the injection site within the subcutaneous fat pads in a region ∼5–10 mm in length and 1–2 mm across, and there was no visible difference in signal area or intensity between the EP voltages tested (Figures 5b–e). At the microscopic cellular level, adipocytes were distinguished by their large diameter (50–100 μm) and characteristic globular shape because of the lipid droplet occupying the center of the cell volume (Figures 5f and g). The fat pads of guinea pigs receiving adipose-EP possessed numerous GFP-expressing adipocytes that were easily distinguishable by their sharp fluorescent outline. In addition, there were regions of strong, diffuse autofluorescence located in the extracellular space between adipocytes, and the collagen septa were also prominently fluorescent. In guinea pigs receiving plasmid DNA injection without adipose-EP, there were no detectable GFP-expressing adipocytes or regions of high autofluorescence, and the collagen septa were visible, but less prominent.

**Figure 6:**
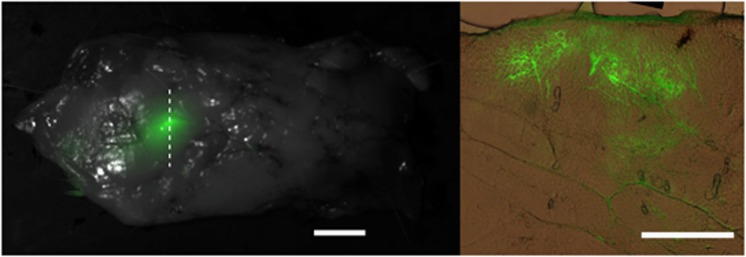
(Left) An intact guinea fat pad was extracted 3 days following adipose-EP at 200 V, showing localized GFP expression at the EP site with the dorsal surface visible. This tissue was used for further histological analysis, and the dotted line indicates the sectioning plane. (Right) A histological section from the same sample, cut 100 μm thick in the transverse plane, with the membrane at the outer dorsal surface indicated (M). GFP (green) is overlaid with brightfield color image of unstained tissue. Scale bars are 10 mm (left) and 1 mm (right).

Further histological analysis was performed to visualize the distribution of reporter construct through the depth of the fat pad. Again, the strongest and most abundant GFP signal was localized to adipocytes adjacent to the collagenous septa dividing the adipose lobes (Figure 6, right). No GFP was detectable in the overlying skin layer (not shown). Gene expression was detectable several millimeters deep into the fat, and was generally consistent with fluid distribution observed in dye injection studies.

**Figure 7:**
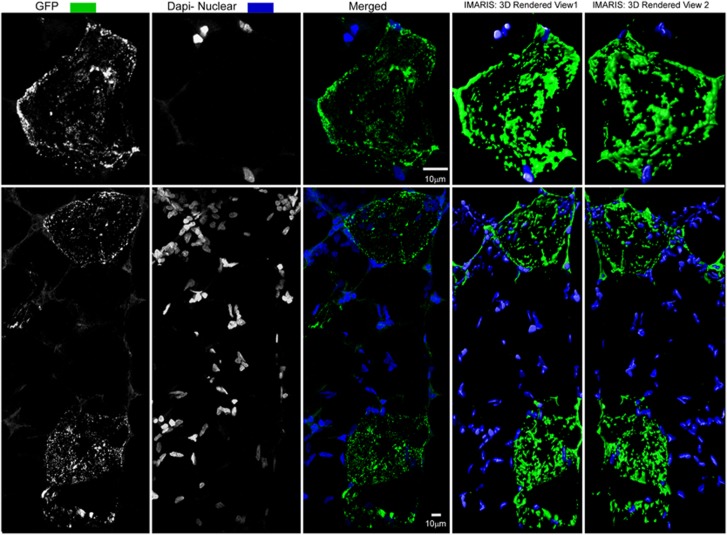
Confocal image showing GFP expression (green) and nuclei (blue) in a single focal plane (middle column) and two different three-dimensional (3D) perspectives (right two columns).

High-resolution confocal images revealed that GFP was expressed in a distinct punctate manner surrounding each transfected adipocyte (Figure 7). GFP expression was not associated with the numerous nuclei surrounding and in between the adipocytes that are indicative of a smaller, secondary cell population within adipose. This population is known to include preadipocytes, fibroblasts and endothelial cells.

### Gene expression kinetics and histological analysis

**Figure 8:**
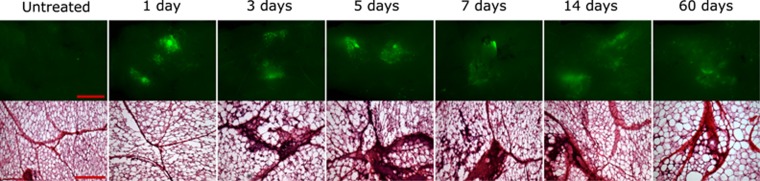
Gene expression kinetics and histological changes following adipose-EP at 200 V. Scale bars for GFP expression (top) are 10 mm, and scale bars for hematoxylin and eosin (H&E)-stained sections (bottom) are 200 μm.

To investigate the kinetics of reporter construct expression in an adipocyte population, guinea pig fat pads were removed, sectioned and analyzed at defined time points following 200 V adipose-EP using noninvasive plate electrodes. Gene expression was measurable as early as 24 h following adipose-EP treatment, and expression was sustained throughout the 60 days monitored (Figure 8, top). Qualitatively, there was no clear difference in the intensity or distribution of the GFP fluorescence over the first 7 days. The signal appeared more diffuse beginning at day 14, and even weaker and more diffuse at day 60. Each distinct site of GFP expression was on the order of 10 mm in diameter.

Histological changes following adipose-EP, as observed through hematoxylin and eosin staining of adipose sections, were noticeable beginning at day 3, continued through day 14 and appeared to mostly resolve by day 60 (Figure 8, bottom). No detectable difference in tissue physiology at 3 h (not shown) or 24 h post treatment was observed. At these early time points, adipocytes were well defined, lipid storage droplets were identifiable as empty voids and collagenous septa were visible because of darker eosin staining and numerous nuclei. Beginning at day 3 and persisting through the length of the 60 days of observation, collagenous septa at the treatment site were noticeably more prominent, likely because of the visualization of large numbers of nuclei from infiltrating cells. In regions where the collagenous septa were more prominent, the extracellular space around adipocytes became populated with higher numbers of cells as well. These histological changes were most prominent between 3 and 7 days post treatment. By 60 days post treatment, cellular infiltration into the extracellular space was mild and the cell density within the collagenous septa was still elevated, but less pronounced.

### Humoral immunogenicity

Although expression of reporter gene constructs in adipose tissue was an extremely promising observation, the applicability of this technology for DNA vaccination would only be relevant if that expression was able to drive an immune response. To assess this, guinea pigs were immunized with a construct expressing the influenza A nucleoprotein (PR8) antigen using adipose-EP, with ID-EP for comparison, and binding titers were measured using enzyme-linked immunosorbent assay (ELISA). The adipose-EP experimental groups included high-voltage EP with 1 injection site (HV-1), high-voltage EP with 5 injection sites (HV-5), low-voltage EP with 1 injection site (LV-1) and low-voltage EP with 5 injection sites (LV-5). All guinea pigs received the same total DNA dose.

**Figure 9:**
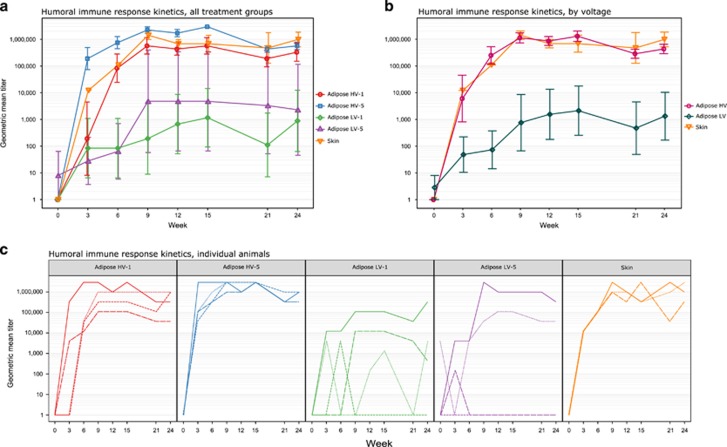
Guinea pig antibody response to adipose-EP and ID-EP vaccination with plasmid DNA encoding influenza A nucleoprotein (PR8) antigen. Guinea pigs were vaccinated at weeks 0, 3, 6 and 21 with 25 μg plasmid. (**a**) Humoral immunogenicity kinetics of different adipose-EP treatment methods in guinea pigs, with ID-EP (skin) for comparison (*n*=4). (**b**) The same immunogenicity data, grouped by EP voltage (*n*=8 for adipose HV and adipose LV, *n*=4 for skin). (**c**) Titers of individual animals, separated by treatment group, with line types indicating different biological replicates. Data are geometric mean titer±s.e. (**a**, **b**) or individual end point titer (**c**). Adipose-EP treatment parameters are abbreviated as HV=high voltage (200 V) and LV=low voltage (50 V), and for (**a**), the number of DNA injection sites is indicated by a number (1 or 5).

HV adipose-EP and ID-EP resulted in similar antibody response kinetics and magnitude, but LV adipose-EP treatments resulted in highly variable and generally lower antibody responses compared with HV adipose-EP or ID-EP (Figures 9a–c). Within HV adipose-EP, 5 injections appeared to generate faster onset of immune response than a single injection site. For LV-treated adipose-EP guinea pigs, there were guinea pigs in both groups that had low or nondetectable titers throughout the study. There were main effects of voltage (F(1, 99)=65.16, *P*=1.68 × 10^−12^), time (F(1, 99)=5.32, *P*=0.023) and number of injection sites (F(1, 99)=4.47, *P*=0.037) on titers of animals receiving adipose-EP. There was no significant interaction between any of these factors.

Because voltage appeared to have a stronger effect upon adipose-EP titers than number of treatment sites, grouped analysis was performed to compare the immunogenicity of ID-EP, HV adipose-EP and LV adipose-EP (Figure 9b). There were main effects on titers due to both treatment group (F(2, 122)=43.31, *P*=6.13 × 10^−15^) and time (F(2, 122)=7.20, *P*=0.0083), with no interaction (F(2, 122)=0.035, *P*=0.97). Although not necessary because of the lack of interaction, simple main effects analysis was also performed. This analysis indicated that the titer difference between HV and LV adipose-EP treatments was significant from week 6 onward (0.00048<*P*<0.018). ID-EP also provided generally higher titers than LV adipose-EP from week 6 onward (0.013<*P*<0.092), and there was no difference in titers between HV adipose-EP and ID-EP at any time point (0.93<*P*<1).

### Cellular immunogenicity

The results from the above study demonstrated the ability of delivery of a DNA vaccine, delivered into adipose tissue via EP, to induce robust humoral responses. To investigate the cellular arm of the immune response, Enzyme-Linked ImmunoSpot (ELISpot) was performed on peripheral blood from immunized guinea pigs. HV-1 (*n*=3), HV-5 (*n*=2) and ID-EP (*n*=2) had fewer replicates as a result of low viable cell counts. Because of the low number of replicates for this assay, we were most interested in observing trends, and although we present statistical analysis, we caution against interpretations of significance.

**Figure 10:**
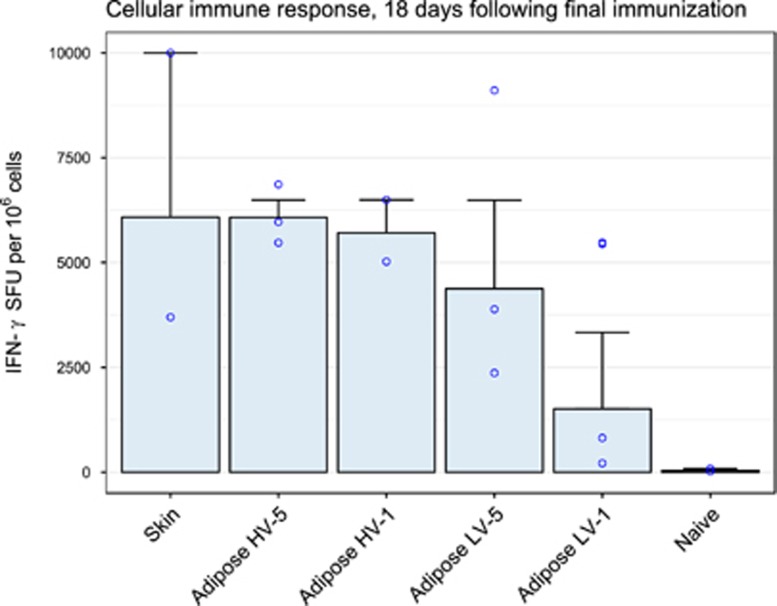
Guinea pig T-cell immune response to adipose-EP and ID-EP vaccination with plasmid DNA encoding influenza A nucleoprotein (PR8) antigen. Guinea pigs were vaccinated at weeks 0, 3, 6 and 21 with 25 μg plasmid, and ELISpot was performed 18 days following final immunization. Results are shown for peptide pool 1. Treatments groups are divided by EP site (skin or adipose), and adipose-EP treatments are further divided by voltage (HV=200 V, LV=50 V) and number of plasmid injection sites (1 or 5). Data are geometric mean±s.e.

All vaccinated guinea pigs produced interferon-γ in response to all three peptide pools, as well as Concanavalin A (not shown). Peptide pool 1, which was the most immunogenic, was used for further analysis (Figure 10). Spot counts appeared similar between ID-EP and HV adipose-EP groups, and trended lower for LV adipose-EP, with LV-1 in particular appearing to elicit the weakest cellular immune response. Within adipose-EP treatment groups, factorial analysis of variance (ANOVA) revealed no significant difference in log-transformed spot counts for voltage (*P*=0.15) or number of injection sites (*P*=0.26), and there was no interaction between these two factors (*P*=0.39). One-way ANOVA comparison of all treatment groups, including ID-EP, showed no significant difference in log-transformed spot counts (*P*=0.31).

## Discussion

Historically, the majority of *in vivo* EPs, both preclinically and clinically, have been performed in skeletal muscle. The combination of DNA delivery with IM-EP has led to a strong body of data demonstrating the capability of the technology to significantly enhance the uptake of DNA to mammalian cells *in vivo*, vastly improve the resulting immune responses and most importantly induce clinical efficacy through disease regression. However, IM-EP, although highly applicable for specific disease targets, remains an invasive procedure associated with some transient pain.^24, 25, 26, 27^ In a bid to address these challenges, significant advances have recently been made in the optimization of EP devices targeting skin.^25, 28^ These advances have led to improvements in the invasiveness, the tolerability and the overall general acceptability of EP technology as a prophylactic vaccination solution.

In this study, we extend that conceptual development by investigating adipose as a target tissue for EP-enhanced DNA vaccination. Adipose tissue has typically been viewed as an inert organ of lipid storage. However, adipose as a target tissue for EP and DNA vaccination may possess favorable characteristics compared with skin and muscle. Although all humans have subcutaneous fat, Western populations, who are severely impacted by obesity rates, have a somewhat unlimited and highly accessible number of treatment sites,^29^ whereas their skeletal muscle is correspondingly more difficult to access. Adipose tissue has low levels of innervation,^30^ leading to a potentially more tolerable EP treatment site. Adipocytes are long-lived cells, and the low turnover rate permits persistence of expressed signal.^31^ Because of their paracrine and endocrine functions, adipocytes are able to produce and secrete proteins at high levels.^20, 32, 33^ And finally, although this has only recently been investigated, subcutaneous fat appears to play an immunomodulatory role, with significant number of immune cells residing in fat tissue and adipocytes themselves possessing the ability to act as antigen-presenting cells.^21, 22^

When evaluating these stand-alone observations together, it made sense to investigate adipose as a possible target for EP-enhanced DNA vaccination. Although others have investigated EP in fat tissue to visualize protein production, they utilized an invasive technique requiring the surgical opening of skin flaps to expose the underlying subcutaneous fat, and then placed the electrodes directly onto the adipose tissue.^34^ The authors believe that this is the first documentation of a noninvasive, clinically applicable technique demonstrating the relevance of adipose as a target tissue for DNA-based immunizations.

### Finite element analysis

Finite element analysis suggested that noninvasive plate electrodes preferentially concentrate the electric field into adipose tissue, whereas needle electrodes provide the same electric field indiscriminately to each tissue they penetrate. In addition, the field generated by plate electrodes is more uniform compared with needle electrodes. These results are consistent with previous calculations used to model EP of other tissue types such as skin, muscle or liver.^35, 36, 37^

The finite element model assumed constant electrical conductivity for each tissue type. Recent evidence suggests that conductivity is in fact a function of electric field strength, and therefore changes dynamically during EP.^38^ However, these dynamic models have only been validated in skin, muscle and tumor tissue, and hence we chose to use constant conductivity to avoid making any assumptions that incorrectly modify the electric field distribution within adipose. Furthermore, we wanted to avoid overestimating the insulating capacity of adipose tissue, and hence we created a model with a very thin adipose layer directly below the bottom edge of the electrodes, permitting current flow through underlying muscle. In reality, the underlying fat is likely much thicker even with the electrodes firmly in place. Therefore, the results of our simulations can be considered a ‘worst case’ scenario, and *in vivo* validation of a dynamic conductivity model in adipose should improve the accuracy our predictions. We believe that an optimized prototype based on noninvasive electrodes could easily be applied clinically and that the data generated in this finite element analysis could be extrapolated to the variable adipose thicknesses found in humans.

### Dye injections and gene expression

Although dye studies suggested that some injectate is in contact with the dermis, no gene expression was observed outside of the fat pad (not shown). This observation is consistent with the finite element analysis that suggests that electric fields of sufficient strength that cause transfection are almost exclusively generated in the adipose tissue. When multiple injections were performed, some sites were more prominent than others, and this may be because of nearby injection sites merging when they are squeezed between the plate electrodes. The strongest dye staining occurred along the collagen septa dividing adipose lobes and, indeed, many transfected adipocytes were clustered around these septa. We hypothesize that both the electric current and the DNA solution primarily travel via these collagenous septa, and adipose transfection occurs when DNA solution escapes these channels and comes into contact with nearby adipocytes before EP. A more refined computer model that includes these low-resistance collagen septa will allow our future simulations to more accurately predict electric field distribution within the tissue, and may help explain the clusters of transfected adipocytes observed. Future studies are underway to improve the distribution of fluid with the adipocyte tissues.

On a cellular level, the treatments appeared highly selective for adipocytes, because despite the numerous other cell types occupying space between adipocytes, GFP was only observed around adipocytes. This is likely because of the propensity of EP to preferentially transfect larger cells at lower field strengths. Adipocytes are globular cells with very large diameter (50–100 μm), and hence their shape and diameter make them more susceptible to EP than other, smaller cell types.^39^ A more thorough investigation into direct transfection of nonadipocytes in fat would have to be carried out to definitively demonstrate selectivity, however.

### Gene expression kinetics

Adipose tissue was shown to be capable of rapid and sustained gene expression after a single adipose-EP treatment. Interestingly, there were no histological signs of cellular infiltration until 3 days post treatment, even though gene expression was robust as early as 24 h after treatment. However, these kinetics may differ for a highly immunogenic antigen, rather than GFP. Previously, it was reported that small numbers of adipocytes (20–60 cells) can be selectively transfected *in vivo* by directly applying EP to surgically exposed adipose tissue using forceps electrodes with ∼0.065 cm^2^ contact surface area.^34^ Here, a noninvasive EP technique is used to transfect large numbers of adipocytes using plate electrodes with ∼100 times the surface area.

### Immunogenicity

The humoral immune response following adipose EP DNA vaccinations was shown to be dependent upon both voltage and spatial distribution of DNA, with higher voltage in particular being critical to achieve rapid, high-magnitude antibody responses. To our knowledge, this is the first demonstration that transfected adipocytes can elicit an immune response. It was surprising to see such strong voltage dependence for humoral immunogenicity, as we saw no voltage-dependent differences in gene expression. The positive impact of multiple treatment sites was anticipated, as more cells are in contact with plasmid. The authors do not believe this to be a clinical hurdle as designing a device that targets multiple sites simultaneously has been previously tested by this group for skin vaccination.^40^

Adipose-EP was capable of producing equivalent cellular immune responses to ID-EP, and though the differences between groups were not significant because of low replicates and high variability, the spot counts trended lower for those guinea pigs receiving the lowest voltage and only one treatment site. These findings appear to support the dependence of adipose-EP immunogenicity upon both EP voltage and DNA distribution, and they suggest that electroporation parameters and DNA distribution within tissue are important factors that can independently be tuned to improve immune responses.

The explanation for the voltage dependence of the antibody response has two key factors. First, higher voltages produce a larger, stronger electric field, and hence more cells can potentially be transfected. Transfection efficiency and immune responses have been shown to be voltage dependent in other tissues such as skin and muscle.^35, 36, 41^ However, the results of our optimization studies indicated that ample transfection was occurring even at low voltages, and hence this is unlikely to be the only explanation. The second explanation for the voltage-dependent antibody response is that higher voltages require higher electric current that can cause tissue damage or irritation because of resistive heating, and it has previously been suggested that electrical energy from EP has an adjuvant effect.^42, 43, 44, 45^ Therefore, it is possible that the cellular infiltration observed is linked to the mild thermal damage caused by EP, and plays a role in the increased immune response at high voltages. It should be noted that although 200 V is similar to the voltages used in IM DNA EP vaccinations, the current never exceeded 1 A that is also similar to IM-EP. Therefore, a similar amount of electrical energy is spread over a larger surface area (6.25 cm^2^) compared with a typical IM-EP setup using two 19 mm, 22-gauge needles spaced 10 mm apart (0.88 cm^2^), and hence the energy density at the electrode surface should be approximately sevenfold lower in the adipose DNA EP treatment compared with IM-EP. ID-EP devices have highly variable electrode configurations, but a general case using 3 mm, 26-gauge needles spaced 5 mm apart at 100 V would still generate nearly sixfold higher energy densities than adipose-EP, even though they operate at lower voltages and currents because of the closely spaced needles.

The mild positive effect upon immunogenicity because of increasing number of DNA injection sites can likely be explained by the increased number of cells in contact with DNA before EP. We demonstrated that there is no detectable benefit to gene expression by increasing injection volume at a single site beyond 50 μl (data not shown), and hence we chose to distribute the same dose of DNA over 5 different sites, each receiving 50 μl injections. The fact that multi-site treatments may provide an immunogenicity benefit compared with single-site, same-dose treatment, particularly at early time points, provides evidence that adipose EP DNA vaccination can directly benefit by exposing more adipocytes to DNA.

Taken together, our results suggest that the immune response can be amplified by involving more adipocytes and providing optimal electroporation parameters. Moving forward, it will be important to characterize how these two factors interact in order to establish a standard treatment protocol. In addition, other factors such as pulse duration, number of pulses, interpulse delay and DNA concentration may all contribute to the immune response and merit further investigation. Finally, the treatments were performed with standard plate electrodes. For future investigation of adipose-EP, it would be beneficial to develop a device that more efficiently focuses the electric field into adipose for a variety of patient body types.

## Materials and methods

### Animals

All animal studies were performed under protocols approved by an institutional animal care and use committee. Female Hartley guinea pigs ∼16 weeks old were used for all *in vivo* studies. Treatments and blood collections were performed while animals were maintained under general anesthesia by inhaled isoflurane. Subcutaneous injections were performed into the interscapular subcutaneous fat pads (located at the scruff of the neck) using a 29-gauge insulin needle oriented parallel to the spine. For terminal studies, guinea pigs were first placed under general anesthesia and then humanely killed by intracardiac injection of pentobarbital.

### Finite element analysis

To simulate different EP modalities, two tissue models were created as 3D CAD assemblies in SolidWorks 2013 (SolidWorks Corp, Concord, MA, USA). Both models consisted of three electrically isotropic layers: skin (1 mm thick), adipose (5 mm thick) and muscle (>10 mm thick). The stratum corneum was not included in these models because voltages as low as 50 V will permeabilize the stratum corneum within microseconds, and its contribution to total tissue resistance then becomes negligible.^46, 47^ Furthermore, the thickness of the stratum corneum is on the order of 20 μm, and very thin layers can cause artifacts in finite element analysis. To model tissue clamped between plate electrodes, two square plate electrode geometries with rounded edges and contact area measuring 4 cm^2^ were placed on opposite sides of a tissue fold comprising two skin layers, two adipose layers and a small 1 mm muscle layer separating a portion of the two adipose layers (Figure 1a). To model penetrating needle electrodes, two 22-gauge needle geometries were placed into the flat tissue geometry with an interelectrode spacing of 10 mm and a penetration depth of 18 mm (Figure 1b).

The two tissue-electrode assemblies were exported to ANSYS Maxwell 2015.2 (ANSYS Software, Canonsburg, PA, USA) for finite element analysis. Electrical conductivity values for each tissue type were based on literature values,^48^ and were assumed to be constant. Conductivity values and general tissue dimensions used in the models are listed in Table 1. A built-in adaptive meshing algorithm was used to generate the mesh used for analysis. An excitation voltage was assigned to one electrode, whereas the opposing electrode was assigned a voltage of zero, and cross-sections bisecting the electrodes in the *x*–*y* analysis plane were created to visualize the electric field distribution.

### Plasmids

Gene expression studies utilized plasmid DNA encoding GFP. Immune studies were carried out using plasmid DNA encoding full-length nucleoprotein from Influenza A (H1N1, A/Puerto Rico/ 8). All plasmid formulations were prepared in saline sodium citrate buffer for a final buffer concentration of 1 ×.

### Dye injection studies

Methylene blue (Sigma-Aldrich, St Louis, MO, USA) was dissolved in deionized water at a concentration of 0.5 mg ml^−1^. For single-site injections, guinea pigs were injected subcutaneously with 100 μl of methylene blue solution. For multi-site injections, five separate 50 μl subcutaneous injections were performed, spaced ∼5 mm apart. Following injection, the entire fat pad was gripped tightly between two plate electrodes to simulate the full treatment protocol. Animals were immediately killed and the fat pads were imaged intact, and then dissected along the sagittal plane and imaged again to visualize dye distribution within the tissue.

### General adipose-EP treatment procedure

Treatment sites were shaved and cleaned. Adipose-EP treatments were performed on the subcutaneous fat pad in the interscapular region, whereas skin treatments were performed on the flank. Immediately following DNA injection, two plate electrodes attached to opposing caliper jaws were coated with conductive gel and then used to pinch the tissue surrounding the injection site, and pulses were administered using the Elgen 1000 control unit (Inovio Pharmaceuticals, San Diego, CA, USA). For ID-EP treatments, DNA was injected intradermally followed immediately by electroporation using the surface electroporation device consisting of a 4 × 4 array of needle electrodes.^15^

### Gross imaging and histological analysis

Following adipose-EP using GFP plasmid, intact fat pads were harvested at predetermined time points and imaged using a FluorChem R imaging system (ProteinSimple, San Jose, CA, USA). Then, fat pads were frozen, and samples measuring ∼10 mm × 10 mm were cut from the transfected region of the fat pad and cryosectioned at a thickness of 30 μm either along the transverse plane to view the depth of transfection or along the coronal plane to view the horizontal distribution of transfected cells. Some sections were fixed in 4% formalin, cleared in xylene, stained with either 4',6-diamidino-2-phenylindole or Hoechst 3342 (Life Technologies, Carlsbad, CA, USA), and coverslipped using Fluoromount (eBioscience, San Diego, CA, USA). Other sections were fixed in formalin, cleared in xylene, stained with hematoxylin and eosin and coverslipped using Permount (VWR, Radnor, PA, USA). Sections that were hematoxylin and eosin stained were imaged in brightfield using an Olympus BX51 microscope (Olympus, Center Valley, PA, USA) equipped with a MicroPublisher 3.3 camera (QImaging, Surrey, BC, Canada). Fluorescence images were captured with a Retiga 3000 camera (QImaging). Confocal images were acquired as high-resolution, multi-paneled and auto-stitched *z*-stacks of the whole tissue using a Zeiss LSM 780 laser scanning confocal microscope (Carl Zeiss, Jena, Germany) and the images were further processed using Zen 2012 (Carl Zeiss) and IMARIS software (Bitplane, Belfast, UK).

### GFP expression and cellular kinetics

Adipose-EP was performed on 14 guinea pigs, using 100 μg of a plasmid coding for GFP and EP parameters of 200 V, 3 pulses, 100 ms duration and 100 ms interpulse delay. As controls, two guinea pigs were treated with EP but did not receive plasmid injection, whereas two additional guinea pigs received the plasmid injection but were not treated with EP. Controls were killed 3 days following treatments, and treated guinea pigs were killed at intervals (*n*=2) following the treatment, ranging from 3 h to 14 days post treatment, as well as a long-term follow-up at day 60. Fat pads were imaged intact for GFP expression and then sectioned and stained with hematoxylin and eosin to visualize signs of cellular infiltration at the treatment site.

### Immunogenicity study

Guinea pigs were treated with 25 μg of nucleoprotein plasmid. Four groups of guinea pigs (*n*=4) received adipose EP treatments as described above, and each group received either a single 100 μl DNA injection or five separate 50 μl injections. Plasmid injection was immediately followed by a single EP treatment consisting of three 100 ms square wave pulses, 200 ms interpulse delay and voltage of either 50 or 200 V. Guinea pigs vaccinated via ID-EP with the surface electroporation device (*n*=3) served as a comparator group for this study as this method has been previously shown to transfect epidermal cells but not subcutaneous adipocytes.^15^ The four adipose-EP groups were as follows: HV-1, HV-5, LV-1 and LV-5. For guinea pigs receiving 5 injections, a single EP procedure was performed immediately following the final injection. The total dose of plasmid DNA was identical for all groups. The study design is illustrated in Table 2.

Every 3 weeks for the duration of the study, 300 μl of blood was collected and serum was stored at −20 °C until analysis. Immunizations were administered at weeks 0, 3, 6 and 21. At 18 days following the final immunization, 3 ml of blood was collected and peripheral blood mononuclear cells were separated for ELISpot analysis.

### ELISA

Serum from vaccinated guinea pigs was analyzed using ELISA. ELISAs were performed using 96-well plates (Thermo Fisher Scientific, Waltham, MA, USA) coated overnight with 100 μl per well of 0.3 μg ml^−1^ nucleoprotein antigen (Sino Biological, Beijing, China) in Dulbecco’s phosphate-buffered saline (VWR). Plates were washed, blocked with phosphate-buffered saline containing 3% bovine serum albumin (Sigma-Aldrich) and 0.05% Tween-20 (Sigma-Aldrich) at 150 μl per well for 1 h at 37 °C, and then washed again. Serum was serially diluted from 1:50 to 1:2 952 450 in phosphate-buffered saline containing 1% bovine serum albumin and 0.05% Tween-20 (sample dilution buffer) at 100 μl per well and incubated for 2 h at 37 °C. Plates were then washed, and horseradish peroxidase-conjugated goat anti-guinea pig IgG (Sigma-Aldrich, catalog no. A7289) was diluted 1:10 000 with sample dilution buffer and added to each well at 100 μl per well for 1 h at 37 °C. Plates were washed and tetramethylbenzidine substrate solution (VWR) was added to each well at 100 μl per well and the color development was stopped with tetramethylbenzidine stop reagent solution (VWR) after 6 min. Absorbance values at 450 nm in each well were measured using a SpectraMax PLUS 384 plate reader (Molecular Devices, Sunnyvale, CA, USA), and the cutoff for a positive titer was calculated using the method described by Frey *et al.*,^49^ in which the mean absorbance and s.d. of the negative controls—in this case, the prebleed samples—were used to calculate cutoff absorbance values. End point titers were used for all ELISA results presented.

### ELISpot

At 18 days following the final immunization, 3 ml peripheral blood was drawn and collected in EDTA tubes to perform interferon-γ ELISpot, using methods previously developed in-house. The blood was diluted 1:1 with Hanks’ balanced salt solution and centrifuged over Ficoll-Paque Plus (GE Healthcare Biosciences, Pittsburgh, PA, USA). The buffy coat was harvested and resuspended at a concentration of 1 × 10^6^ live cells per ml in R10 medium, and plated at a density of 1 × 10^5^ cells per well on 96-well Millipore IP plates (MilliporeSigma, Burlington, MA, USA) that had been coated overnight with 5 μg ml^−1^ mouse monoclonal anti-guinea pig interferon-γ antibody (V-E4, a gift from Hubert Schäfer, Berlin, Germany) and blocked with 1 × phosphate-buffered saline containing 10% (w/v) sucrose and 2% (w/v) bovine serum albumin. In triplicate, peripheral blood mononuclear cells were incubated for 18 h with either Concanavalin A or one of three different nucleoprotein antigen peptide pools previously found to be immunostimulatory.^50^ Following a wash to remove cells, 0.2 μg biotinylated mouse monoclonal anti-guinea pig interferon-γ antibody (N-G3, a gift from Hubert Schäfer) was added to each well and allowed to incubate for 2 h. Wells were then washed and 100 μl BCIP/NBT detection reagent substrate was added to each well for 15 min. Plates were imaged using a CTL-Immunospot S6 ELISpot plate reader (Cellular Technology Limited, Cleveland, OH, USA), and CTL-Immunospot software (Cellular Technology Limited) was used to process and count the spots. For each animal, spot counts were normalized by subtracting the counts of unstimulated cells.

### Statistical methods

All animal studies were performed once and the number of biological replicates per group were based on availability, and studies were not designed to detect a prespecified effect size as this work is exploratory in nature. For immunogenicity studies, all guinea pigs were born and received on the same date, and cage mates received the same treatments. The investigators were not blinded during these studies. Because of the small sample sizes in the immune study (*n*=3 or *n*=4), tests of normality are uninformative and parametric statistical tests were performed on log-transformed ELISA and ELISpot data.

To compare ELISA titer data of adipose-EP-treated groups, factorial ANOVA was performed, using EP voltage, number of injection sites and study week as factors. For grouped comparison between adipose-EP at different voltages and ID-EP, two-way factorial ANOVA was performed with treatment group and study week as factors. For specific comparisons of ELISA titer data between all treatments, data were stratified by study week and then one-way ANOVA was performed, and multiple comparisons were made using Tukey’s *post hoc* testing when the F-test was significant. Because this simple main effect analysis at each time point was meant to generate hypotheses for future testing, we sought to minimize type II error and did not correct for multiple comparisons because of the repeated Tukey’s tests across multiple time points.

ELISpot data were analyzed first within adipose-EP-treated groups using factorial ANOVA, with EP voltage and number of treatment sites as factors. One-way ANOVA was performed to compare ELISA data for all treatment groups, including ID-EP. The cutoff for significance was defined as *P*<0.05, and all observations of nonsignificant trends and differences were accompanied by *P*-values. All plots of ELISA and ELISpot data are expressed as geometric mean±s.e.

## Conclusions

The work here demonstrates that an adipose-targeted DNA vaccine is immunogenic following optimization of DNA delivery and electroporation parameters. This approach provides rapid and sustained immune responses, and does not require invasive needle electrodes. At a fixed dose of DNA, the magnitude and onset of the immune response both improve with electroporation voltage and increasing number of injection sites. Adipose-targeted EP DNA vaccination offers potential safety, tolerability and ease-of-use advantages over IM administration and does not suffer from the dosage or cell turnover limitations of ID treatments. As such, the authors believe that this platform warrants further investigation.

## Figures and Tables

**Table 1:** Finite element analysis model parameters and tissue model layer thicknesses used in simulations

*Material*	*Conductivity, S m*^*−1*^	*Thickness, mm*
Skin	0.25	1
Adipose	0.05	5
Muscle	0.3	>10
Stainless steel	1.1x10^6^	NA

Abbreviation: NA, not available.

**Table 2:** Immunology study design for EP DNA vaccination of adipose tissue

*Group*	n	*Treatment site*	*Injections*	*Voltage, V*	*Total volume, μl*	*Total DNA dose, μg*
HV-1	4	Adipose	1	200	100	25
LV-1	4	Adipose	1	50	100	25
LV-5	4	Adipose	5	50	250	25
HV-5	4	Adipose	5	200	250	25
ID-EP	3	Skin	1	25	50	25

Abbreviations: EP, electroporation; HV-1, high-voltage EP with 1 injection site; HV-5, high voltage EP with 5 injection sites; ID-EP, intradermal EP; LV-1, low-voltage EP with 1 injection site; LV-5, low-voltage EP with 5 injection sites.

All EP treatments were performed using 3 pulses with 100 ms duration and 200 ms interpulse delay. Adipose-EP was performed with caliper-type electrodes, and ID-EP was performed with a specialized needle array designed to target skin.

## References

[bib1] Ugen KE, Nyland SB, Boyer JD, Vidal C, Lera L, Rasheid S et al. DNA vaccination with HIV-1 expressing constructs elicits immune responses in humans. Vaccine 1998; 16: 1818–1821. 9795386 10.1016/s0264-410x(98)00180-7

[bib2] Le TP, Coonan KM, Hedstrom RC, Charoenvit Y, Sedegah M, Epstein JE et al. Safety, tolerability and humoral immune responses after intramuscular administration of a malaria DNA vaccine to healthy adult volunteers. Vaccine 2000; 18: 1893–1901. 10699338 10.1016/s0264-410x(99)00407-7

[bib3] MacGregor RR, Boyer JD, Ugen KE, Lacy KE, Gluckman SJ, Bagarazzi ML et al. First human trial of a DNA-based vaccine for treatment of human immunodeficiency virus type 1 infection: safety and host response. J Infect Dis 1998; 178: 92–100. 9652427 10.1086/515613

[bib4] Mir LM, Bureau MF, Rangara R, Schwartz B, Scherman D. Long-term, high level *in vivo* gene expression after electric pulse-mediated gene transfer into skeletal muscle. Comptes Rendus l’Académie des Sci Ser III Sci la Vie 1998; 321: 893–899. 10.1016/s0764-4469(99)80003-1 9879468

[bib5] Mathiesen I. Electropermeabilization of skeletal muscle enhances gene transfer *in vivo*. Gene Therapy 1999; 6: 508–514. 10476210 10.1038/sj.gt.3300847

[bib6] Low L, Mander A, McCann K, Dearnaley D, Tjelle T, Mathiesen I et al. DNA vaccination with electroporation induces increased antibody responses in patients with prostate cancer. Hum Gene Ther 2009; 20: 1269–1278. 19619001 10.1089/hum.2009.067

[bib7] Tsang C, Babiuk S, van Drunen Littel-van den Hurk S, Babiuk LA, Griebel P. A single DNA immunization in combination with electroporation prolongs the primary immune response and maintains immune memory for six months. Vaccine 2007; 25: 5485–5494. 17408815 10.1016/j.vaccine.2007.03.009

[bib8] Livingston BD, Little SF, Luxembourg A, Ellefsen B, Hannaman D. Comparative performance of a licensed anthrax vaccine versus electroporation based delivery of a PA encoding DNA vaccine in rhesus macaques. Vaccine 2010; 28: 1056–1061. 19896452 10.1016/j.vaccine.2009.10.111

[bib9] Weiland O, Ahlén G, Diepolder H, Jung M-C, Levander S, Fons M et al. Therapeutic DNA vaccination using *in vivo* electroporation followed by standard of care therapy in patients with genotype 1 chronic hepatitis C. Mol Ther 2013; 21: 1796–1805. 23752314 10.1038/mt.2013.119 PMC3776630

[bib10] Yin J, Dai A, Lecureux J, Arango T, Kutzler MA, Yan J et al. High antibody and cellular responses induced to HIV-1 clade C envelope following DNA vaccines delivered by electroporation. Vaccine 2011; 29: 6763–6770. 21195801 10.1016/j.vaccine.2010.12.055 PMC10839813

[bib11] Best SR, Peng S, Juang C-M, Hung C-F, Hannaman D, Saunders JR et al. Administration of HPV DNA vaccine via electroporation elicits the strongest CD8+ T cell immune responses compared to intramuscular injection and intradermal gene gun delivery. Vaccine 2009; 27: 5450–5459. 19622402 10.1016/j.vaccine.2009.07.005 PMC2745985

[bib12] Bagarazzi ML, Yan J, Morrow MP, Shen X, Parker RL, Lee JC et al. Immunotherapy against HPV16/18 generates potent TH1 and cytotoxic cellular immune responses. Sci Transl Med 2012; 4: 155ra138. 10.1126/scitranslmed.3004414 PMC4317299 23052295

[bib13] Trimble CL, Morrow MP, Kraynyak KA, Shen X, Dallas M, Yan J et al. Safety, efficacy, and immunogenicity of VGX-3100, a therapeutic synthetic DNA vaccine targeting human papillomavirus 16 and 18 E6 and E7 proteins for cervical intraepithelial neoplasia 2/3: a randomised, double-blind, placebo-controlled phase 2b trial. Lancet 2015; 386: 2078–2088. 26386540 10.1016/S0140-6736(15)00239-1 PMC4888059

[bib14] Smith TR, Schultheis K, Kiosses WB, Amante DH, Mendoza JM, Stone JC et al. DNA vaccination strategy targets epidermal dendritic cells, initiating their migration and induction of a host immune response. Mol Ther Methods Clin Dev 2014; 1: 14054. 26052522 10.1038/mtm.2014.54 PMC4448738

[bib15] Broderick KE, Shen X, Soderholm J, Lin F, McCoy J, Khan AS et al. Prototype development and preclinical immunogenicity analysis of a novel minimally invasive electroporation device. Gene Therapy 2011; 18: 258–265. 20962869 10.1038/gt.2010.137

[bib16] Hirao LA, Wu L, Khan AS, Satishchandran A, Draghia-Akli R, Weiner DB. Intradermal/subcutaneous immunization by electroporation improves plasmid vaccine delivery and potency in pigs and rhesus macaques. Vaccine 2008; 26: 440–448. 18082294 10.1016/j.vaccine.2007.10.041

[bib17] Hooper JW, Golden JW, Ferro AM, King AD. Smallpox DNA vaccine delivered by novel skin electroporation device protects mice against intranasal poxvirus challenge. Vaccine 2007; 25: 1814–1823. 17240007 10.1016/j.vaccine.2006.11.017 PMC9628994

[bib18] Zhou Q, Jin J, Zhu L, Chen M, Xu H, Wang H et al. The optimal choice of medication administration route regarding intravenous, intramuscular, and subcutaneous injection. Patient Prefer Adherence 2015; 9: 923. 26170642 10.2147/PPA.S87271 PMC4494621

[bib19] Kershaw EE, Flier JS. Adipose tissue as an endocrine organ. J Clin Endocrinol Metab 2004; 89: 2548–2556. 15181022 10.1210/jc.2004-0395

[bib20] Trayhurn P, Beattie JH. Physiological role of adipose tissue: white adipose tissue as an endocrine and secretory organ. Proc Nutr Soc 2001; 60: 329–339. 11681807 10.1079/pns200194

[bib21] Morris DL, Cho KW, Delproposto JL, Oatmen KE, Geletka LM, Martinez-Santibanez G et al. Adipose tissue macrophages function as antigen-presenting cells and regulate adipose tissue CD4+ T cells in mice. Diabetes 2013; 62: 2762–2772. 23493569 10.2337/db12-1404 PMC3717880

[bib22] Xiao L, Yang X, Lin Y, Li S, Jiang J, Qian S et al. Large adipocytes function as antigen-presenting cells to activate CD4+ T cells via upregulating MHCII in obesity. Int J Obes 2016; 40: 112–120. 10.1038/ijo.2015.145 PMC4722243 26248660

[bib23] Amante DH, Smith TRF, Mendoza JM, Schultheis K, McCoy JR, Khan AS et al. Skin transfection patterns and expression kinetics of electroporation-enhanced plasmid delivery using the CELLECTRA-3P, a portable next-generation dermal electroporation device. Hum Gene Ther Methods 2015; 26: 134–146. 26222896 10.1089/hgtb.2015.020 PMC5206769

[bib24] Kjeken R, Tjelle TE, Kvale D, Mathiesen I. Clinical evaluation of pain and muscle damage induced by electroporation of skeletal muscle in humans. Mol Ther 2004; 9: 60.

[bib25] Diehl MC, Lee JC, Daniels SE, Tebas P, Khan AS, Giffear M et al. Tolerability of intramuscular and intradermal delivery by CELLECTRA(®) adaptive constant current electroporation device in healthy volunteers. Hum Vaccin Immunother 2013; 9: 2246–2252. 24051434 10.4161/hv.24702 PMC3906411

[bib26] Wallace M, Evans B, Woods S, Mogg R, Zhang L, Finnefrock AC et al. Tolerability of two sequential electroporation treatments using MedPulser DNA delivery system (DDS) in healthy adults. Mol Ther 2009; 17: 922–928. 19277016 10.1038/mt.2009.27 PMC2835142

[bib27] Spearman P, Mulligan M, Anderson EJ, Shane AL, Stephens K, Gibson T et al. A phase 1, randomized, controlled dose-escalation study of EP-1300 polyepitope DNA vaccine against Plasmodium falciparum malaria administered via electroporation. Vaccine 2016; 34: 5571–5578. 27697302 10.1016/j.vaccine.2016.09.041 PMC5075504

[bib28] El-Kamary SS, Billington M, Deitz S, Colby E, Rhinehart H, Wu Y et al. Safety and tolerability of the Easy Vax^TM^ clinical epidermal electroporation system in healthy adults. Mol Ther 2012; 20: 214–220. 22068424 10.1038/mt.2011.235 PMC3255586

[bib29] Wang Y, Beydoun MA, Liang L, Caballero B, Kumanyika SK. Will all Americans become overweight or obese? estimating the progression and cost of the US obesity epidemic. Obesity (Silver Spring) 2008; 16: 2323–2330. 18719634 10.1038/oby.2008.351

[bib30] Frayn KN, Karpe F, Fielding BA, Macdonald IA, Coppack SW. Integrative physiology of human adipose tissue. Int J Obes Relat Metab Disord 2003; 27: 875–888. 12861227 10.1038/sj.ijo.0802326

[bib31] Spalding KL, Arner E, Westermark PO, Bernard S, Buchholz BA, Bergmann O et al. Dynamics of fat cell turnover in humans. Nature 2008; 453: 783–787. 18454136 10.1038/nature06902

[bib32] Kershaw EE, Flier JS. Adipose tissue as an endocrine organ. J Clin Endocrinol Metab 2004; 89: 2548–2556. 15181022 10.1210/jc.2004-0395

[bib33] Mohamed-Ali V, Pinkney JH, Coppack SW. Adipose tissue as an endocrine and paracrine organ. Int J Obes Relat Metab Disord 1998; 22: 1145–1158. 9877249 10.1038/sj.ijo.0800770

[bib34] Granneman JG, Li P, Lu Y, Tilak J. Seeing the trees in the forest: selective electroporation of adipocytes within adipose tissue. Am J Physiol Endocrinol Metab 2004; 287: E574–E582. 15126244 10.1152/ajpendo.00567.2003

[bib35] Gehl J, Sørensen TH, Nielsen K, Raskmark P, Nielsen SL, Skovsgaard T et al. *In vivo* electroporation of skeletal muscle: threshold, efficacy and relation to electric field distribution. Biochim Biophys Acta 1999; 1428: 233–240. 10434041 10.1016/s0304-4165(99)00094-x

[bib36] André FM, Gehl J, Sersa G, Préat V, Hojman P, Eriksen J et al. Efficiency of high- and low-voltage pulse combinations for gene electrotransfer in muscle, liver, tumor, and skin. Hum Gene Ther 2008; 19: 1261–1271. 19866490 10.1089/hum.2008.060

[bib37] Miklavcic D, Semrov D, Mekid H, Mir LM. A validated model of *in vivo* electric field distribution in tissues for electrochemotherapy and for DNA electrotransfer for gene therapy. Biochim Biophys Acta 2000; 1523: 73–83. 11099860 10.1016/s0304-4165(00)00101-x

[bib38] Sel D, Cukjati D, Batiuskaite D, Slivnik T, Mir LM, Miklavcic D. Sequential finite element model of tissue electropermeabilization. IEEE Trans Biomed Eng 2005; 52: 816–827. 15887531 10.1109/TBME.2005.845212

[bib39] Agarwal A, Zudans I, Weber EA, Olofsson J, Orwar O, Weber SG. Effect of cell size and shape on single-cell electroporation. Anal Chem 2007; 79: 3589–3596. 17444611 10.1021/ac062049e PMC2532982

[bib40] McCoy JR, Mendoza JM, Spik KW, Badger C, Gomez AF, Schmaljohn CS et al. A multi-head intradermal electroporation device allows for tailored and increased dose DNA vaccine delivery to the skin. Hum Vaccin Immunother 2015; 11: 746–754. 25839221 10.4161/21645515.2014.978223 PMC4514302

[bib41] Aihara H, Miyazaki J. Gene transfer into muscle by electroporation *in vivo*. Nat Biotechnol 1998; 16: 867–870. 9743122 10.1038/nbt0998-867

[bib42] Liu J, Kjeken R, Mathiesen I, Barouch DH. Recruitment of antigen-presenting cells to the site of inoculation and augmentation of human immunodeficiency virus type 1 DNA vaccine immunogenicity by *in vivo* electroporation. J Virol 2008; 82: 5643–5649. 18353952 10.1128/JVI.02564-07 PMC2395223

[bib43] Davalos RV, Rubinsky B, Mir LM. Theoretical analysis of the thermal effects during *in vivo* tissue electroporation. Bioelectrochemistry 2003; 61: 99–107. 14642915 10.1016/j.bioelechem.2003.07.001

[bib44] Chiarella P, Massi E, De Robertis M, Sibilio A, Parrella P, Fazio VM et al. Electroporation of skeletal muscle induces danger signal release and antigen-presenting cell recruitment independently of DNA vaccine administration. Expert Opin Biol Ther 2008; 8: 1645–1657. 18847301 10.1517/14712598.8.11.1645

[bib45] Babiuk S, Baca-Estrada ME, Foldvari M, Middleton DM, Rabussay D, Widera G et al. Increased gene expression and inflammatory cell infiltration caused by electroporation are both important for improving the efficacy of DNA vaccines. J Biotechnol 2004; 110: 1–10. 15099900 10.1016/j.jbiotec.2004.01.015

[bib46] Chizmadzhev YA, Indenbom AV, Kuzmin PI, Galichenko S V, Weaver JC, Potts RO. Electrical properties of skin at moderate voltages: contribution of appendageal macropores. Biophys J 1998; 74: 843–856. 9533696 10.1016/S0006-3495(98)74008-1 PMC1302564

[bib47] Pliquett U, Weaver JCPassive electrical properties of human stratum corneum during application of electric fields. In: Bersani F (Ed.). Electricity and Magnetism in Biology and Medicine. Springer US: Boston, MA, 1999, pp 259–262.

[bib48] Miklavčič D, Pavšelj N, Hart FX, Miklavčič D, Pavšelj N, Hart FXElectric properties of tissues. In: Akay M (ed.). Wiley Encyclopedia of Biomedical Engineering. John Wiley & Sons, Inc.: Hoboken, NJ, USA, 2006.

[bib49] Frey A, Di Canzio J, Zurakowski D. A statistically defined endpoint titer determination method for immunoassays. J Immunol Methods 1998; 221: 35–41. 9894896 10.1016/s0022-1759(98)00170-7

[bib50] Schultheis K, Schaefer H, Yung BS, Oh J, Muthumani K, Humeau L et al. Characterization of guinea pig T cell responses elicited after EP-assisted delivery of DNA vaccines to the skin. Vaccine 2017; 35: 61–70. 27894716 10.1016/j.vaccine.2016.11.052 PMC5221502

